# 

**Published:** 1901-02

**Authors:** 


### PERSONALS.

Dr. J. C. Foelker, of Allentown, Pa., was a visitor to Philadelphia in January and a caller at the JOURNAL office. With sixty-five years to his credit, his interest in veterinary progress is unabated, and he continues a faithful reader of German and English veterinary literature.

Dr. W. C. Rayen, of Nashville, visits the State Capitol almost daily since the Legislature is in session. His mission is to secure legislation for the betterment of the veterinary profession in Tennessee, which we have every reason to believe will be accomplished.

Dr. John J. Millar, city veterinarian, Sioux City, Iowa, has returned from Europe after an extended vacation. While abroad he visited all the principal seats of learning in England, Scotland, France, and Germany.

Dr. G. R. White, D.V.S., Columbian University, class of 1897, is taking a course in human medicine at the University of Nashville in conjunction with his duties with the board of health in the inspection of meat.

President Harger, of the Pennsylvania State Veterinary Medical Association, fully realizes the responsibility of that high trust and has already given the duties a deal of personal attention.

Dr. George P. Statter is sojourning in England, where he expects to remain until the Liverpool Cup is run in April.

Dr. E. M. Ranck, corresponding secretary of the Pennsylvania State Veterinary Medical Association, is an active, energetic worker, and is doing much to win as creditable a record as his worthy predecessors.

The teaching staff of the Grand Rapids Veterinary College is composed of graduates of the Ontario Veterinary College and others who have obtained degrees at the college since its establishment.

Dr. J. E. Foster, of Coshocton, Ohio, health officer for his city, addressed the eleventh annual meeting of the State and local boards of health at Columbus on January 17, 1901.

Dr. Leonard Pearson, State Veterinarian, spent New Year's at the State capital of Pennsylvania, there to witness the opening of the biennial session of the State Legislature.

Dr. William J. Storm, formerly of Dunmore, Pa., is now located in Philadelphia and is in general charge of all the horses of the Philadelphia district of the Atlantic Refining Company.

Dr. Samuel G. Hendren, of the Bureau of Animal Industry, has been transferred from Indianapolis, Ind., to Newark, N. J., and made inspector of the work at that centre.

Dr. David Waugh, of Pittsburg, is now in the British service on the transports from New Orleans with consignments of mules for the South African war.

Dr. Orr Graham, of Port Perry, Ontario, has sold his practice to Dr. J. T. Elliot, of Detroit, Mich., and retires from the veterinary profession.

Dr. Thomas B. Rayner, of Chestnut Hill, Philadelphia, Pa., has been confined to his home for several weeks from a foot affection.

Dr. M. Jacob, of Knoxville, Tenn., is lecturing in the agricultural course at the State College, University of Tennessee, on "Veterinary Science."

Dr. S. B. Willard, of Yardley, Pa., takes an active interest in the local fire department and "runs with the boys" at every alarm sounded.

Dr. Otto Noack, of Reading, was a visitor at the Veterinary Department of the University of Pennsylvania early in January.

Dr. Leonard Pearson, State Veterinarian of Pennsylvania, was a sufferer from tonsillitis early in the new year.

Dr. L. R. Webber, of Rochester, N. Y., conducts a livery and boarding stable in connection with his veterinary hospital.

Dr. F. P. Barker, of Coburn, Pa., is president and treasurer of the Barker Stock Farms, of Centre County.

Dr. Charles H. Perry, of Worcester, Mass., is also the proprietor of one of that city's prominent boarding stables.

Dr. H. D. Fennimore is now located at Los Angeles, Cal., where he will remain during the winter months.

Dr. A. W. Clement, of Baltimore, Md., has been confined to one of Baltimore's hospitals for several weeks, suffering from a complication of diseases ushered in by nervous dyspepsia.

Dr. Leonard Pearson was one of the principal speakers at the meeting of the State Breeders' Association in December.

Dr. Peter I. Kershner, formerly of Manhattan, Kansas, has located at Topeka, Kansas.

Dr. H. B. McDowell, of Middletown, Del., has resumed practice.

Dr. S. J. Wallace, of Warren, Pa., was a visitor to Philadelphia in January.

Dr. W. S. Manley, of Kansas City, Kan., was a visitor to the East during the Christmas holidays, visiting Cincinnati on his return.

